# Correlation of Motor Competence and Social-Emotional Wellbeing in Preschool Children

**DOI:** 10.3389/fpsyg.2022.846520

**Published:** 2022-04-06

**Authors:** Sanja Salaj, Mia Masnjak

**Affiliations:** Motor Development Laboratory, Faculty of Kinesiology, University of Zagreb, Zagreb, Croatia

**Keywords:** skill, development, association, proficiency, movement

## Abstract

**Introduction:**

The relations of motor skills to different developmental domains, i.e., cognitive, emotional, and social domain, are well-documented in research on children with poor motor competence and children with disabilities. Less conclusive evidence on interaction of motor and social or emotional development can be seen in research on typically developing children. The purpose of this study was to determine a correlation between motor skills and social-emotional functioning in typically developing preschool children and to identify differences in social-emotional functioning in children with different levels of motor competence.

**Methods:**

A total of 125 preschool children (67 boys, 58 girls, average age 5.1 years) participated in this study. To assess children’s motor skills, we used the Test of Gross Motor Development–Second Edition that measures locomotor and object-control skills. To screen child’s social and emotional functioning, we used the Ages and Stages Questionnaire–Social Emotional: Second Edition. Spearman’s correlation analysis was used to determine association between motor skills and social-emotional functioning. Difference in social-emotional functioning between groups of preschool children with High and Low motor competences was calculated using Mann-Whitney *U*-test.

**Results:**

The main result of this study is weak correlation of child’s motor skills to social-emotional functioning. Furthermore, preschool children with High and Low motor competences do not differ in risk for social and emotional difficulties.

**Conclusion:**

Further research on typically developing children is needed to have more conclusive evidence on interaction of motor and social or emotional development.

## Introduction

Motor competence is defined as the degree of proficiency of an individual in performing different motor skills (Bardid et al., 2021). Motor competence is positively associated with health-related physical fitness and may help enhance the development of long-term health outcomes in children and adolescents (Cattuzzo et al., 2016). Children with higher motor competence have a lower risk of having body fatness and higher oxygen uptake in future (Lima et al., 2017). Children with negative or reduced positive changes in fitness and motor competence during childhood are at higher risk of being overweight or obese (Rodrigues et al., 2016). A large proportion of children are not able to perform locomotor and object-control skills with proficiency expected for their developmental level (Eather et al., 2018). It is believed that the level of motor skills is a very important and underrated part of the causal mechanism responsible for adult health risk behaviors and physical inactivity (Stodden et al., 2008).

Motor skills can be developed in positive environment combining structured and unstructured physical activities (Dapp et al., 2021). Children with higher level of motor skills tend to be more active and engage in more physical activity than those with poorer motor skills (Williams et al., 2008). In recent studies, there is clear evidence of decline in physical activity every year of child’s life from early childhood onward (Farooq et al., 2020). Reduced physical activity may put skill development, physical fitness, and health at risk.

Emergence of each motor skill lays the foundation for development since it enables new opportunities for learning. With postural control, children can reach and see new parts of the environment; with locomotor skills, they can access new places; and with object-control skills, they can interact with different objects and enhance opportunities for social interaction (Adolph and Hoch, 2020). Thus, adequate motor skills are considered important for child’s overall development, i.e., physical, cognitive, and social-emotional development (Kuzik et al., 2020).

Socio-emotional development in children occurs most often in a predictable order (Cicchetti et al., 1995; Freeman et al., 2014; Martoccio et al., 2014). Recent theories regarding the correlation between emotions and behavior emphasize the fact that the way an individual behaves socially is influenced by emotional experience and expectation because emotion constitutes the primary motivational component for mental and physical action and behavior (Izard, 2009).

The emotional and social wellbeing of preschool children refers to the way they think and feel about themselves and other people. It also comprises the capacity to adapt to daily tasks and issues while maintaining a happy and satisfying life growing up. Within emotional and social development, we must observe the following developmental characteristics and processes: temperament, emotional development in expressing one’s own and understanding other people’s emotions, development of attachment, socio-cognitive development in understanding the concept of self and understanding relationships with others, development of self-regulation, and development of sociability (Starc et al., 2004). The emergence of these socio-emotional skills helps children feel more confident and competent in developing relationships, building friendships, resolving conflicts, persisting when faced with challenges, coping with anger and frustrations, and managing emotions (National Research Council, 2000). A child who is able to establish a relationship with others and is motivated to learn will experience success in school and life.

Emotional development results in the ability to regulate one’s emotions and the impact of emotions on behavior. Children learn through interaction with environment on how to modify and regulate emotions, deal with frustration, encounter danger, and experience fear and anxiety, and as a result, they become successful in interpersonal functioning (LaFreniere, 2000). Good social and emotional functioning provides a solid basis for good adaptation in school, which, in turn, reinforces the child’s sense of belonging, thus correlating with the positive affect, academic self-efficacy, and academic achievement (Nix et al., 2013).

Motor, social-emotional, and cognitive functioning are connected at the neurophysiological level (Cheung et al., 2021). The neural pathways in the central nervous system for motor, social-emotional, and cognitive development overlap and work simultaneously. Regulation of these systems happens through overlapping neural networks in the prefrontal cortex and cerebellum in behavior adjustment (Cheung et al., 2021). Nevertheless, performed movements, manifested motor skills, and how they relate to physical, emotional, social, and cognitive skills are still poorly understood. Some researchers even criticize that the relationship of motor skills and emotional factors such as anxiety is examined more on intuition and clinical experience rather than through systematic research (Piek et al., 2010). Some have well-documented on the relationships of motor skills and different developmental domains, i.e., cognitive, emotional, and social domain, in children with autism spectrum disorders (for review, refer to Ohara et al., 2019), children with developmental disabilities (e.g., specific learning disorder, speech/language impairment, and intellectual disability) (Kim et al., 2016; MacDonald et al., 2017; Cheung et al., 2021), or children with developmental coordination disorder (Emck et al., 2009; Lee et al., 2020). Less conclusive evidence on interaction of motor and social or emotional development can be seen in research on typically developing children. The primary purpose of this study was to determine a correlation between motor skills and social-emotional functioning in typically developing preschool children. The secondary purpose was to determine differences in social-emotional functioning in children with different levels of motor competence.

## Materials and Methods

This research is a part of a national, population-based study on physical activity and motor skills in preschool children (Vukelja et al., 2022). In capital city region, three kindergartens were randomly invited to participate in additional social-emotional screening. The measurements were conducted in two visits to kindergarten during 2 months period. At first visit, parents signed informed consent and filled the questionnaires. At second visit, children’s motor skills were evaluated.

A total of 125 children (67 boys and 58 girls, average age 5.1 years) aged between 5 and 6 years (preschool groups) from public kindergarten participated in this study. Child was included in this study if parent signed the informed consent. All study procedures were approved by the institutional ethical committee (reference number 2014-100). Children were divided into two subgroups based on their level of motor skill, according to the manufacturer’s normative values (Ulrich, 2000): children with gross motor quotient score 89 and below were placed to group Low motor competence (*N* = 37), and children with value 90 and above were placed to group High motor competence (*N* = 88). Sample size for a Spearman’s correlation was determined by using power analysis. The power analysis was conducted (G*Power version 3.1.9.6, Universitat Kiel, Germany) using a two-tailed test, with an alpha of 0.05, a power of 0.80, and a medium effect size of 0.3. As Spearman’s rank correlation coefficient is computationally identical to Pearson’s product-moment coefficient, the power analysis was conducted using software for estimating power of a Pearson’s correlation. Based on the aforementioned assumptions, the required sample size was determined to be 67.

We used the Ages and Stages Questionnaire: Social Emotional: Second Edition (ASQSE) to screen child’s social and emotional functioning (Squires et al., 2015). Parents filled the questionnaires based on their child’s social and emotional behavior in everyday activities. This set of questionnaires assesses functioning and risk based on seven key domains, namely, self-regulation, compliance, adaptive functioning, autonomy, affect, social-communication, and interaction (subdomains include interaction with adults and interaction with peers). Social-emotional components are assessed through partial and overall score that represents the risk: the higher the score, the greater the risk of social-emotional functioning and need for further evaluation and additional follow-up actions. ASQSE was adapted in Croatian language (Masnjak et al., 2016). Reliability of ASQSE is 0.89, the internal validity is 0.84, validity is 0.83, sensitivity is 0.81, and specificity is 0.83 (Squires et al., 2015). Scores are calculated for each of seven domains, two subdomains for interaction with adults and peers and as overall social-emotional score (ASQSE score).

The Test of Gross Motor Development–Second Edition (TGMD2) was used to assess children’s motor skills. TGMD2 was found valid and reliable for use in children aged 3–10 years (Ulrich, 2000). TGMD2 consists of twelve tests, which are divided into two groups of locomotor and manipulative skills. Locomotor tests evaluate running, galloping, hopping, leaping, side-sliding, and jumping for distance. Manipulative tests evaluate efficiency in baseball striking, dribbling, ball catching, kicking, overhead throwing, and underarm rolling. This test showed high-reliability characteristics (Cronbach’s alpha: 0.82–0.94) (Ulrich, 2000). Testing was conducted using the same measurs, with warm-up procedure of 5 min running in circle followed by 2-min dynamic stretching. Each child performed one familiarization trial and two test trials. Movements were recorded and analyzed retrospectively. Each skill is scored, and the raw scores are summed into locomotor and manipulative raw skill score. Raw skill scores are standardized according to age and gender into three variables, namely, standardized locomotor (TGMD2 LOC), manipulative (TGMD2 MAN), and overall motor score or gross motor quotient (TGMD QUOTIENT).

A statistical analysis of study data was carried out using IBM SPSS Statistics for Windows (version 24.0, IBM Corp., Armonk, NY, United States). The Kolmogorov-Smirnov test (KS) was used to check the normality of distribution. All KS values were significant (*p* < 0.01), with ASQSE score being 0.14, self-regulation 0.16, compliance 0.43, adaptive functioning 0.34, autonomy 0.23, affect 0.28, social communication 0.36, interaction with adults 0.25, and interaction with peers 0.30. Since the data were not distributed normally in socio-emotional variables, Spearman’s correlation analysis was used to determine association between motor competence and social-emotional functioning. Based on the work by Dancey and Reidy (2007), Spearman’s correlation coefficients are classified as follows: weak 0.10, moderate 0.40, and strong 0.70. Difference in social-emotional functioning between preschool children with high vs. low level of motor competence was calculated using Mann-Whitney *U*-test. A level of statistical significance was set at *p* < 0.05.

## Results

Mean values and standard deviation of socio-emotional and motor competence variables are presented in Table 1.

**TABLE 1:** Mean values and standard deviation of motor competence and socio-emotional variables.

	Mean	Std. Dev.
ASQSE score	39.34	27.11
Self-regulation	15.04	10.07
Compliance	1.92	3.22
Adaptive functioning	4.12	7.10
Autonomy	5.40	4.94
Affect	3.84	4.72
Social communication	3.28	3.83
Social interaction	6.60	6.97
Social interaction with adults	3.84	4.92
Social interaction with peers	1.80	3.88
TGMD2 LOC	10.32	2.11
TGMD2 MAN	7.90	1.93
TGMD QUOTIENT	94.60	9.58

*Mean, Arithmetic mean; Std. Dev, standard deviation; ASQSE score, overall social-emotional score; TGMD2 LOC, standardized locomotor score; TGMD2 MAN, standardized manipulative score; TGMD QUOTIENT, overall motor score.*

Table 2 presents correlations between the motor competence and social-emotional functioning scores. Results show weak correlations between all variables. A weak significant correlation was found between social interaction and child’s overall (r_s_ = 0.19) and locomotor (r_s_ = 0.21) skills (*p* < 0.05). When children were divided into High and Low motor competence groups based on their overall motor score, the Mann-Whitney *U*-test did not show significant differences in ASQSE variables (Table 3).

**TABLE 2:** Spearman’s correlation coefficients of TGMD2 scores and ASQSE score and ASQSE subdimensions.

	TGMD2 LOC	TGMD2 MAN	TGMD QUOTIENT
ASQSE score	0.07	0.06	0.04
Self-regulation	0.15	−0.04	0.04
Compliance	0.05	−0.09	−0.07
Adaptive functioning	0.03	0.08	0.05
Autonomy	−0.12	0.04	−0.07
Affect	0.08	0.09	0.10
Social communication	0.19*	0.14	0.21*
Social interaction	0.07	0.08	0.07
Social interaction with adults	0.14	0.03	0.10
Social interaction with peers	−0.07	0.06	−0.04

*Significant at p < 0.05.

**TABLE 3:** Mann-Whitney *U*-test in ASQSE variables of high and low motor competence group of preschool children

		Mean Rank	Sum of Ranks	Mann-Whitney *U*	*p*-value
ASQSE score	Low	60.46	2237.00	1534.00	0.61
	High	64.07	5638.00		
Self-regulation	Low	60.00	2220.00	1517.00	0.54
	High	64.26	5655.00		
Compliance	Low	66.03	2443.00	1516.00	0.45
	High	61.73	5432.00		
Adaptive functioning	Low	61.45	2273.50	1570.50	0.72
	High	63.65	5601.50		
Autonomy	Low	66.84	2473.00	1486.00	0.42
	High	61.39	5402.00		
Affect	Low	60.50	2238.50	1535.50	0.59
	High	64.05	5636.50		
Social communication	Low	54.00	1998.00	1295.00	0.04*
	High	66.78	5877.00		
Social interaction	Low	59.95	2218.00	1515.00	0.52
	High	64.28	5657.00		
Social interaction with adults	Low	61.07	2259.50	1556.50	0.67
	High	63.81	5615.50		
Social interaction with peers	Low	64.38	2382.00	1577.00	0.72
	High	62.42	5493.00		

*Significant at p < 0.05.

## Discussion

The main result of this study is weak correlation of child’s motor skills with social-emotional functioning. Furthermore, preschool children with high and low motor competences do not differ in risk for social and emotional difficulties.

Previous research studies on relation of motor skills and other development areas such as social, emotional, cognitive, and speech are dominantly related to children with developmental difficulties and disorders. Piek and associates in a series of paper found motor skill competence was linked to cognitive, social, and emotional functioning (Skinner and Piek, 2001; Piek et al., 2006; Piek et al., 2008). In children with developmental coordination disorder, small-to-moderate correlation was found between motor coordination skills and emotional and behavioral difficulties (Emck et al., 2009; Lee et al., 2020) and moderate correlation between motor coordination skills and anxiety and depression (Piek et al., 2008). In addition, motor skills were found to be related to cognitive skills in children with developmental coordination disorder (Asonitou et al., 2010) and to social and speech skills (Hsu et al., 2004) and adaptive social and communication skills in children with autism (MacDonald et al., 2013). In our study, we found significant but low correlation between overall and locomotor skills and social functioning. Nevertheless, there are some inconsistent findings within population of children with disabilities. Fine motor skills, but not gross motor skills, were related to cognitive and social skills in pre-kindergarten children with developmental disabilities (Kim et al., 2016), and motor skills were related to socialization, communication, and daily living skills in young male, but not female, children with developmental disabilities (MacDonald et al., 2017). On the contrary, we found no significant correlation of interaction with adults or peers and motor skills and weak correlation of communication with motor skills. In a review on association between motor skills and social skills in children with autism spectrum disorders (Ohara et al., 2019), 75% of analyzed studies reported association between overall motor skill scores and social skills. Two of these studies reported that, as compared with gross motor skills, fine motor skills tended to have stronger correlations with social skills. One study reported that fine motor skills were significantly related to adaptive social and communication skills, but no significant correlations between social skills and gross motor skills (MacDonald et al., 2013). In this study, there were low correlations of gross motor skills with social skills. Among fine motor skills, manual dexterity tended to relate most to social skills (Ohara et al., 2019). Fine motor skills and manual dexterity were not measured in our study, while correlation of object-control skills with social skills was small. Not all studies in children with disabilities show consistent relations; one that included children with autism showed motor skills are not related to social interaction (Dadgar et al., 2017). In our study on typically developing preschool children, motor skills were not related to social interaction with adults or peers. Furthermore, in the study by Ecevit and Şahin (2021) on typically developing preschool children, no relevant correlations were found between their motor and social skills. In our study, the difference in communication skills found in comparison of children with high vs. low motor competences suggests that children with high motor competence might prefer body movements to verbal communication. Based on our results, we cannot be conclusive on the interdependence of motor skills and communication and interaction skills of a child.

Although the relationship between different developmental domains shows relatively high level of consistency in children with difficulties, research on typically developing children is lacking. Cheung et al. (2021) reported that both fine and gross motor skills contribute to social-emotional skills in younger and older children with disabilities and in older typically developing children. Also, motor skills of children were related to their academic performance. In children with and without developmental coordination disorder (Lee et al., 2020), correlation values of Movement Assessment Battery for Children test scores and problems in emotional functioning ranged from trivial to moderate, with the highest being depression (*r* = −0.468). The authors concluded that motor coordination difficulties in children cause peer relationship difficulties, mental health problems, and emotional problems such as depression and anxiety. In our study, weak insignificant correlation was found with motor skills and total social-emotional score of subdimensions of emotion, such as self-regulation, compliance, adaptive functioning, autonomy, and affect. Also, children with high and low motor competence did not have different risks for emotional functioning in abovementioned areas. Previous research studies on relationships of motor skills and other developmental domains imply that children with poor motor competence are at greater risk of developing cognitive, speech, social, and emotional difficulties. In our study, children with low motor competence did not have higher risk of emotional or social problems. From the perspective of preschool teachers, this finding has positive result; if poorer motor skills in typically developing children are not related to emotional or social functioning, then developmental delay in one area potentially does not imply delay in other developmental areas. The weak correlation findings in our study may be due to different testing procedures focusing more on gross motor skill rather than fine motor skills and balance.

There is a lack of research on relationship between motor skills and social-emotional behavior in typically developing children and its findings are still contradictory. Researchers argue that focusing on the extreme end of the motor skill continuum might overestimate the relationship between constructs in the general population (Rigoli et al., 2012). More research on typically developing children is needed to have more conclusive evidence on interaction of motor skills and social or emotional development. Most of the correlation analyses were done using large variety of tests for motor competence and/or social-emotional skills. Regarding motor competence testing, there are more than forty different test batteries used around the world. Some of them focus on fine motor skills, other to coordination, and some on combined skill and motor abilities testing. For example, Movement Assessment Battery for Children–Second Edition (Movement ABC-2; Henderson et al., 2007) is constructed to identify, describe, and guide treatment of motor impairment and is composed of manual dexterity, manipulative skills, and balance testing. Bruininks-Oseretsky Test of Motor Proficiency–Second Edition (BOT-2; Bruininks and Bruininks, 2005) is a set of fine motor tests, coordination and balance, speed, agility, and strength, while Test of Gross Motor Development–Second Edition (Ulrich, 2000), which was used in this research, is composed of six locomotor (i.e., running, galloping, hopping, leaping, side-sliding, and jumping for distance) and six manipulative (i.e., baseball striking, dribbling, ball catching, kicking, overhead throwing, and underarm rolling) skills. Therefore, the use of different tests for motor competence and social-emotional functioning is the possible reason for differences in the correlation values.

Motor skills may potentially differ in the developmental trajectories through which cascading changes in social-emotional functioning can occur. Nevertheless, research on relationship between different developmental domains does not imply a causal inference. Randomized controlled trials including motor coordination exercises or sport participation appear to be effective in improving social and behavioral outcomes (Griffiths et al., 2010; Piek et al., 2015). There is a need for preschool-level intervention measures for children who face greater difficulty with movement. For typically developing children, holistic approach to development of motor, social, and emotional skills can be recommended. Common misconception is that motor skills appear in natural manner during early childhood. Compared with cognitive skills, for example, systematic motor skills development is sometimes neglected. Motor skills should be learned and practiced and equally valued as other skills in preschool educational settings. Teachers can apply traditional games and preschool activities in which all developmental domains are targeted and child-oriented approach is nurtured. Development of locomotor and object-control skills in early childhood may contribute to physical activity level later in future. There is consistent evidence that motor competence is associated with increased physical activity levels during the early childhood and adolescent years as well as long-term physical activity and motor skill performance (Loprinzi et al., 2015). Research show that children with higher level of object-control skills have up to 20% higher chance to being physically active in the adolescence (Barnett et al., 2009). Competence in object-control skills is considered a cornerstone of improved future physical activity and healthier lifestyles (Pienaar et al., 2021). Early childhood is the best time to develop motor skills, which will present foundation for lifelong physical activity. Within preschool environment, it is important to promote and organize activities to improve motor skills and everyday physical activity and healthy lifestyle behaviors. These could include a systematic sequence of development of basic motor skills that are organized through play in a way that encourages the social, emotional, and cognitive development of the child.

This study has limitations. Due to the lack of associations between children’s motor and social-emotional skills in our population of children enrolled in kindergarten program (about 60% of all preschool children), we were unable to draw generalized conclusions to population of all preschool-aged children. Specific test batteries for motor skills and social-emotional functioning also contributed to differences in relative size and its significance. Further investigation on the relationship of motor skills and social-emotional functioning in typically developing preschool children is needed.

## Data Availability Statement

The raw data supporting the conclusions of this article will be made available by the authors, without undue reservation.

## Ethics Statement

The studies involving human participants were reviewed and approved by the Scientifical and Ethical Board of Faculty of Kinesiology University of Zagreb. Written informed consent to participate in this study was provided by the participants’ legal guardian/next of kin.

## Author Contributions

MM collected the data and participated in writing methods and introduction section. SS designed the research, revised the manuscript, wrote introduction and discussion, analyzed the data, and wrote the manuscript with MM. Both authors have made a substantial, direct, and intellectual contribution to the work, and approved it for publication.

## Conflict of Interest

The authors declare that the research was conducted in the absence of any commercial or financial relationships that could be construed as a potential conflict of interest.

## Publisher’s Note

All claims expressed in this article are solely those of the authors and do not necessarily represent those of their affiliated organizations, or those of the publisher, the editors and the reviewers. Any product that may be evaluated in this article, or claim that may be made by its manufacturer, is not guaranteed or endorsed by the publisher.
